# Cerclage Wiring Improves Biomechanical Stability in Distal Tibia Spiral Fractures Treated by Intramedullary Nailing

**DOI:** 10.3390/jcm12051770

**Published:** 2023-02-22

**Authors:** Stefan Förch, Sabrina Sandriesser, Christian von Rüden, Edgar Mayr, Peter Augat

**Affiliations:** 1 Department of Trauma, Orthopaedic, Plastic and Hand Surgery, University Hospital of Augsburg, Stenglinstrasse 2, 86156 Augsburg, Germany; 2 Institute for Biomechanics, BG Unfallklinik Murnau, Prof. Küntscher Str. 8, 82418 Murnau, Germany; 3 Institute for Biomechanics, Paracelsus Medical University, Strubergasse 21, 5020 Salzburg, Austria; 4 Department of Trauma Surgery, BG Unfallklinik Murnau, Prof. Küntscher Str. 8, 82418 Murnau, Germany

**Keywords:** cable, cerclage, tibia shaft, comminuted fracture, intramedullary nailing, biomechanics

## Abstract

Background: Partial weight-bearing after operatively treated fractures has been the standard of care over the past decades. Recent studies report on better rehabilitation and faster return to daily life in case of immediate weight-bearing as tolerated. To allow early weight-bearing, osteosynthesis needs to provide sufficient mechanical stability. The purpose of this study was to investigate the stabilizing benefits of additive cerclage wiring in combination with intramedullary nailing of distal tibia fractures. Methods: In 14 synthetic tibiae, a reproducible distal spiral fracture was treated by intramedullary nailing. In half of the samples, the fracture was further stabilized by additional cerclage wiring. Under clinically relevant partial and full weight-bearing loads the samples were biomechanically tested and axial construct stiffness as well as interfragmentary movements were assessed. Subsequently, a 5 mm fracture gap was created to simulate insufficient reduction, and tests were repeated. Results: Intramedullary nails offer already high axial stability. Thus, axial construct stiffness cannot be significantly enhanced by an additive cerclage (2858 ± 958 N/mm NailOnly vs. 3727 ± 793 N/mm Nail + Cable; *p* = 0.089). Under full weight-bearing loads, additive cerclage wiring in well-reduced fractures significantly reduced shear (*p* = 0.002) and torsional movements (*p* = 0.013) and showed similar low movements as under partial weight-bearing (shear 0.3 mm, *p* = 0.073; torsion 1.1°, *p* = 0.085). In contrast, additional cerclage had no stabilizing effect in large fracture gaps. Conclusions: In well-reduced spiral fractures of the distal tibia, the construct stability of intramedullary nailing can be further increased by additional cerclage wiring. From a biomechanical point of view, augmentation of the primary implant reduced shear movement sufficiently to allow immediate weight-bearing as tolerated. Especially, elderly patients would benefit from early post-operative mobilization, which allows for accelerated rehabilitation and a faster return to daily activities.

## 1. Introduction

Fractures of the tibial shaft represent the most common long bone fractures [1]. After operative fracture fixation, partial weight-bearing is still considered the post-operative treatment of choice according to the AO surgery reference [2]. However, the recent literature supports the need for a shift in the post-operative weight-bearing regimen toward early mobilization [3,4]. Especially in the geriatric patient population, immediate weight-bearing as tolerated is becoming more prevalent and is treated as one of the key elements for successful rehabilitation [5]. Thus, immediate loading of the treated limb implies the need for increased implant stability.

In combination with a distal tibia locking plate, additive cable cerclage wiring has already been proven from a biomechanical aspect, to increase construct stability and allow for immediate post-operative weight-bearing [6,7]. Promising first clinical results emphasize the beneficial stabilizing effect of supplemental cerclage wiring in distal tibia spiral fractures [8]. A minimally invasive technique guarantees a careful and safe cerclage insertion with only 3% of cerclages inducing local tissue irritation [8]. Moreover, a recent literature review could not find any direct link between cerclage wiring on the periosteal blood supply and delayed or inhibited fracture healing [9].

In extra-articular tibia fractures, intramedullary nailing offers a suitable alternative that ensures satisfactory clinical outcomes [10]. Nailing seems slightly superior in terms of post-operative complications and infection rates [11], but appears to be associated with higher malunion rates [10]. To achieve sufficient fracture reduction and to stabilize a torsional fracture of the tibia, the use of cerclage wires has already been suggested [8,12,13]. Nonetheless, the stabilizing effect of cerclage wiring in combination with a tibia nail in a realistic fracture model has not yet been investigated in biomechanical studies.

Thus, the aim of this biomechanical study was to investigate additional cerclage wiring in combination with intramedullary nailing for the fixation of distal tibia spiral fractures. We hypothesized that in a well-reduced fracture, an additional cable cerclage will reduce interfragmentary movements under full weight-bearing conditions. Furthermore, we assumed that additional cerclage wiring has a limited stabilizing effect at a larger fracture gap or comminuted fracture zone.

## 2. Materials and Methods

For this biomechanical study, a spiral shaft fracture (AO/OTA 42-A1.1c) was cut with the help of a custom-made sawing template at the distal third of synthetic composite tibiae (large left, fourth generation, Sawbones Europe AB, Malmoe, Sweden). A total number of fourteen samples were reproducibly fractured and instrumented with a standard tibia nail (T2 tibia nail standard, ø 11 × 390 mm, Stryker GmbH & Co. KG, Duisburg, Germany) by using another template for fragment reposition. Implantation was conducted by an experienced trauma surgeon and led to a complete reduction of the fracture gap in all samples. Distally, the nail was locked freehand by placing all three screw options. At the proximal tibia, two screws were placed via the targeting device, and the most proximal screw was omitted.

**Figure 1:**
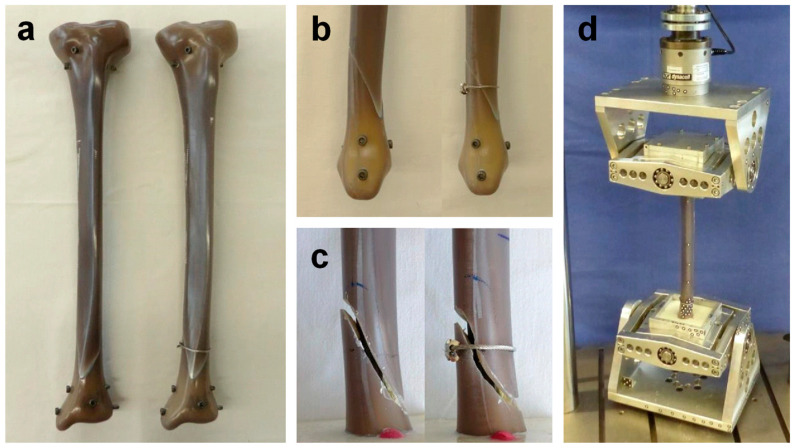
Synthetic tibia sample and test setup: (**a**) frontal view on the instrumented tibia with the solitary nail and with supplemental cable cerclage wiring around the fracture zone; (**b**) medial view on the distal tibia with a reduced fracture gap; (**c**) 5 mm fracture gap; (**d**) test setup with two cardan joints to avoid constraining forces.

Half of the samples were tested as solitary nail fixation (*n* = 7 NailOnly) and in the other half the fracture was further stabilized by a supplemental steel cable cerclage (*n* = 7 Nail + Cable) (ø 1.7 mm, DePuy Synthes Companies, Oberdorf, Switzerland) looped around the fracture zone (Figure 1a). According to the manufacturer’s recommendation, the cerclage was tightened under a tension of 50 kg and closed by a crimp mechanism.

Prior to mechanical testing the tibiae were aligned vertically and were embedded in polyurethane (RenCast FC 53 A/B + filler DT 082, Huntsman, The Woodlands, TX, USA) at both ends to achieve a resulting working length of 295 mm. To avoid embedding the implant, the nail entry point as well as the screw heads and the slightly protruding screw tips were covered with modeling clay.

Mechanical testing was performed on a servo-hydraulic testing machine (Instron 8874, Dynacell, measuring range ± 10 kN, accuracy ± 2% and ±100 Nm, accuracy ±1%, Instron Structural Testing GmbH, High Wycombe, UK) with cardan joints to avoid constraining forces (Figure 1d). The load protocol covered clinically relevant partial (20 kg) as well as full (75 kg) weight-bearing loads and was adopted from previous studies [6,7]. To settle each construct, an axial sinusoidal load of 10–200 N at a frequency of 1 Hz was applied for a total of 100 cycles, followed by a pure axial ramp up to 200 N at a velocity of 0.1 mm/s to determine initial axial construct stiffness by analyzing the linear portion of the force–displacement curve.

The first part of the load protocol consisted of quasi-static testing under combined axial and torsional loads of approximately 20 kg partial (200 N and 2 Nm) and 75 kg full weight-bearing loads (750 N and 7 Nm). In this specific test setup, the applied torsion mimicked internal rotation.

After quasi-static testing, each sample underwent a dynamic load protocol to simulate clinically relevant post-operative loading. Torsional loading was applied at a frequency of 0.5 Hz, alternating between ±4 Nm. Axial sinusoidal loading was applied at 1 Hz, starting between 50 N (valley) and 200 N (peak), and peak load increased by 50 N after every 1000 cycles. Tests were terminated when reaching a load maximum of 2000 N.

Finally, to mimic a more complex, comminuted, or incompletely reduced fracture condition, an interfragmentary gap of 5 mm was created in each construct by manually grinding material along the fracture line (Figure 1c). To investigate imperfect cerclage tension, the cable cerclage was kept in place and has not been replaced. The same quasi-static tests under partial and full weight-bearing loads were conducted and the results were compared to the well-reduced samples.

To determine interfragmentary movements, small adhesive marker points were attached along the fracture line on the proximal and distal fragments and these points were tracked by an optical 3D motion tracking system (ARAMIS Professional 5 M, GOM GmbH, Braunschweig, Germany). For quasi-static testing pictures were taken at each unloaded and loaded state and for dynamic testing movements at the maximum load of 2000 N were analyzed. Translational and rotational movements in the fracture gap were evaluated. The coordinate system was aligned in a way that the vertical axis was oriented along the tibial shaft axis and defined axial movement. Sagittal and frontal axes were defined according to the respective anatomical orientations. Shear movements were defined as movement in the transverse plane. Rotational movements were calculated as rotation around the tibial shaft axis and sagittal axis. To guarantee reproducible data processing, the origin of the coordinate system was placed at the same position for all specimens.

Axial construct stiffness was calculated by dividing the force by the deformation along the vertical shaft axis. Axial, shear, and rotational movements were assessed at partial (200 N), full (750 N), and maximum (2000 N) loading. For statistical analysis, data were tested for normal distribution using Shapiro–Wilk tests. Axial stiffness was statistically compared using unpaired t-tests. For quasi-static loading, the reduced condition was compared to the gap condition using Wilcoxon tests for paired samples, and Mann–Whitney tests for unpaired comparisons with and without additive cerclage. For dynamic loading, the NailOnly group was compared to the Nail + Cable group using unpaired *t*-tests (SPSS Statistics, Version 26, IBM, Armonk, NY, USA). Values are given as mean and standard deviation.

## 3. Results

Solitary nail fixation already achieved high axial construct stiffness and could not be significantly increased by a supplemental cable cerclage (2858 ± 958 N/mm NailOnly vs. 3727 ± 793 N/mm Nail + Cable; *p* = 0.089). The 5 mm gap condition reduced axial stiffness for NailOnly (1283 ± 538 N/mm; *p* = 0.003) as well as for Nail + Cable (1028 ± 271 N/mm; *p* < 0.001).

**Figure 2:**
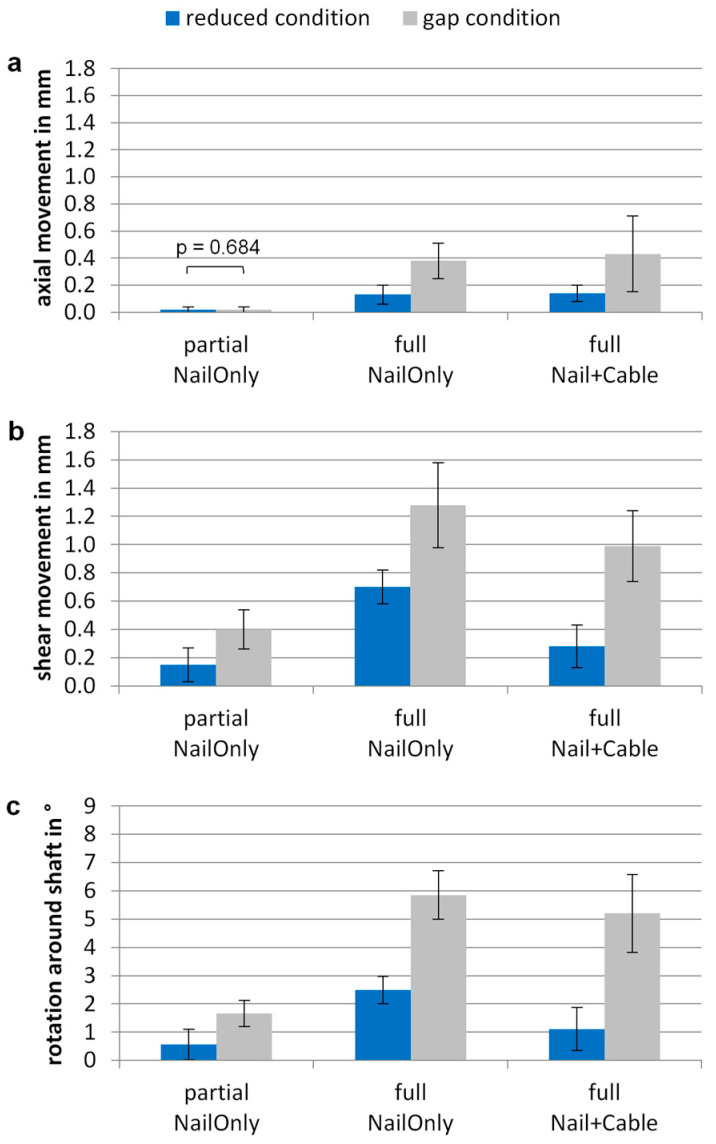
Interfragmentary motion under quasi-static partial and full weight-bearing loads for a well-reduced fracture condition and for a gap condition. Differences between NailOnly group and Nail + Cable group are shown for (**a**) axial movement in mm; (**b**) shear movement in mm and (**c**) rotation around the shaft axis in °. Values are given as mean ± standard deviation. Movements in the gap condition were significantly larger (*p* ≤ 0.018) compared to the reduced condition, if not otherwise indicated.

Under quasi-static loading, well-reduced constructs showed significantly less interfragmentary movement (*p* ≤ 0.018) compared to constructs with remaining gap condition for all loading scenarios, except for axial movement under partial weight-bearing showing no movement at all (*p* = 0.684) (Figure 2).

Under full weight-bearing, axial movement remained below 0.2 mm and 0.5 mm for reduced condition and gap condition, respectively. Both cases showed little axial movements with no further stabilization by additional cerclage wiring.

In the well-reduced fracture condition, shear movement amounted to 0.7 ± 0.1 mm under full weight-bearing. Additional cerclage wiring significantly reduced shear movement to 0.3 ± 0.1 mm (*p* = 0.002), which was comparable to the shear movement under partial weight-bearing without cable (0.2 ± 0.1 mm; *p* = 0.073). For the gap condition, shear movement increased to 1.3 ± 0.3 mm and could not be reduced by an additive cable cerclage (1.0 ± 0.3 mm; *p* = 0.073).

The highest rotation around the shaft axis was observed for the NailOnly group under full weight-bearing, with 2.5 ± 0.5° for the reduced condition and up to 230% higher rotations for the gap condition (5.9 ± 0.9°). In the reduced condition, the addition of a cable cerclage significantly restricted rotations to 1.1 ± 0.8° (*p* = 0.013), which was comparable to partial weight-bearing (*p* = 0.085). In the gap condition, no further reduction was achieved by additional cerclage wiring.

**Figure 3:**
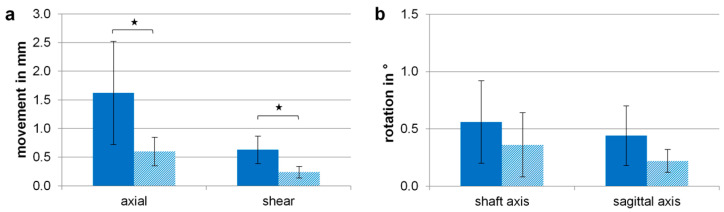
Resulting motion for the reduced fracture condition at maximum applied load of 2000 N and 4 Nm after dynamic loading of the NailOnly (solid bar) and the Nail + Cable (dashed bar) groups for (**a**) axial and shear movements and (**b**) rotational movements around the shaft axis and the sagittal axis. Values are given as mean ± standard deviation and significant differences are marked by the asterisk symbol (* *p* < 0.05).

All samples survived dynamic loading up to 2000 N without any construct failure. The cerclage wiring significantly reduced translational movements by 63% for axial (*p* = 0.032) and by 62% for shear movements (*p* = 0.006) (Figure 3). Rotational movements were generally at a low level of less than 0.5°. With an additional cable cerclage, rotations were reduced by 36% around the shaft axis (*p* = 0.315) and by 50% around the sagittal axis (*p* = 0.086), but without statistical significance.

## 4. Discussion

Distal tibia shaft fractures can be reliably stabilized by intramedullary nailing. In this study, it has been demonstrated that for well-reduced spiral fractures, the stabilization can be further increased by applying cerclage wiring around the fracture zone. The additional stabilization especially reduced shear movements at the fracture site, which is considered of particular importance for an undisturbed fracture healing process. The extent of added construct stiffness and movement reduction suggests that spiral fractures of the distal tibia are to be allowed for immediate weight-bearing as tolerated without the risk of construct failure, and loss of reduction of malalignment.

As interfragmentary movements play a crucial role in callus formation and fracture healing, a supplemental cerclage serves not only as a temporary reduction tool during nail insertion but improves the overall stability of the fracture fixation [14,15]. The results of the present study show that only in well-reduced fractures the cerclage provides increased construct stability. Larger fracture gaps of 5 mm simulating a comminuted fracture zone, resulted in significantly higher movements, which could not be reduced by the addition of a cable cerclage. The reported results underline the importance of a good reduction for the stability of osteosynthesis. In clinical practice, the insertion of a cerclage for spiral fractures often allows anatomical reduction.

Depending on the localization and type of fracture, a cerclage has no stabilizing effect in comminuted or transverse fracture patterns but develops its potential in spiral and oblique fractures [16]. In other orthopedic and trauma surgeries, e.g., the femoral shaft, additional cerclage wires experience broad approval and contribute to the overall stability of the osteosynthesis [17,18,19,20].

Only a few studies support the use of additional cerclage wiring in combination with intramedullary nailing for the stabilization of spiral fractures of the distal tibia. A recent study was published promising clinical results on 96 tibia shaft spiral fractures treated with additive cerclages to increase the stability of the osteosynthesis [8]. Huang et al. reported on effective and simplified fracture reduction in oblique and spiral fractures and improved fixation stability due to additional cerclage wiring [13]. Habernek published 37 cases of torsional tibia fractures treated by intramedullary nailing and percutaneous cerclage wiring, already more than 30 years ago [12]. In addition to the advantages in fracture reduction, he reported on early full weight-bearing and benefits in fracture healing. However, the concept of early weight-bearing did not gain acceptance and the recommendation of partial weight-bearing prevailed.

In recent years, more and more literature emerged, questioning the restrictions in post-operative weight-bearing and initiating a discussion towards early loading as tolerated [3,5]. It was found that, especially in elderly patients, immediate mobilization reduced post-operative complications, led to successful rehabilitation, and improved the overall outcome after hip fractures [3,5,21,22] as well as after fractures of the distal femur [4,23]. A randomized controlled trial investigating 115 patients after surgically treating ankle fractures revealed that immediate mobilization and weight-bearing as tolerated led to faster return to work and improved functional outcomes [24]. According to Gross et al. immediate weight-bearing after intramedullary nailing of isolated distal tibia shaft fractures (AO/OTA type 42-A and 42-B) is not related to complications or adverse events [25]. To our knowledge, this randomized controlled trial is the only study investigating a comparable fracture type to the present study (AO/OTA 42-A1.1c) and supporting the concept of early loading. However, all these mentioned studies examine the effect of immediate weight-bearing from a clinical perspective. Our present study investigates for the first time the biomechanical performance, including construct stiffness and interfragmentary motion, of distal tibia fractures treated by intramedullary nailing and supplemental cerclage wiring.

Depending on fracture height and involvement of the ankle joint, fractures at the distal tibia can also be treated by plate osteosynthesis. A previous study showed that in combination with a distal tibia locking plate, an additive cerclage has been proven to increase overall construct stability to allow for immediate weight-bearing, from a biomechanical point of view [6]. Investigating the same fracture model, the current findings reveal increased axial construct stiffness, irrespective of the use of an additive cerclage. Due to the principle of load transfer, intramedullary implants show higher axial stiffness compared to extramedullary locking plates [26]. In our study, axial stiffness ranges between 2800 and 3700 N/mm, which is within the favorable range above 2500 N/mm for fracture gaps smaller than 3 mm [27].

Accordingly, for fracture gaps smaller than 3 mm, interfragmentary movements of 0.2 to 1.0 mm offer perfect conditions for bone healing [28]. Thus, micromotions in the fracture gap seem favorable for callus formation and fracture healing [28]. In a study by Epari et al., a clear relationship was found between the stability of osteosynthesis and the mechanical strength of the healing bone [29]. It is therefore concluded that moderate degrees of axial stability are related to a higher callus strength [29]. These findings are true for axial stability. However, sufficient reduction of shear movements is equally important for bone healing [14]. From a clinical point of view, increasing the stability of the fracture fixation allows for an earlier and less restricted mobilization of the patient as compared to fractures with insufficient reduction or imperfect alignment. Alignment, reduction, and stabilization result in load sharing between osteosynthesis implant and bone and have been shown to result in a more favorable healing outcome [30,31].

Relating to clinical aspects, nailing and plating of extra-articular distal tibia fractures show both satisfactory results [10]. Nailing seems slightly superior in terms of post-operative complications, infection rates, and reduced surgery time, but poses the risk of higher malunion rates [11,32,33,34,35]. To reduce the risk of malunion, proper alignment, and sufficient fracture reduction can be achieved by an additional cerclage looped around the fracture zone. Our study demonstrates that an additive cerclage cannot increase axial construct stiffness, but significantly lowers shear movements in the fracture gap. The current literature agrees on the fact that satisfactory surgical treatment of tibia shaft fractures is challenging to achieve and implant selection should be carefully made for each individual patient [10].

Implant flexibility and as a consequence the amount of interfragmentary movement, especially shear movement in the transverse plane, strongly depends on the diameter of the intramedullary nail [36]. It is therefore recommended to use thicker nail diameters in order to achieve adequate implant stability. Sufficient reduction of shear movements is also essential to trigger the onset of callus formation and to achieve adequate fracture healing [14,29]. Another study confirmed that good reduction of the fragments with small fracture gaps promotes healing and induces good revascularization [37]. Nonetheless, the blood supply and tissue irritation at distal tibia fractures still remain the subject of controversial discussion, especially when using additive cerclage wires. Even though there is only little soft tissue covering the lower third of the tibia, no correlation between impaired healing and cerclage wiring directly on the periosteum was found in a clinical study reporting on the first promising results [8]. The radially oriented blood vessels are not disrupted by cerclage wiring when following a minimally invasive approach and tissue-preserving implantation [16]. A careful cerclage insertion is of particular importance in elderly patients with reduced bone quality.

Limitations of this study include the inherent weakness of biomechanical in vitro studies to not represent in vivo situations and healing processes. Instead of human specimens, synthetic bone models have been used to exclude inter-specimen variability and to focus on implant fixation and the stabilizing effect of additive cerclage wiring [38]. Cut-through or failure of the cerclage could not be induced in these synthetic bones and was not the subject of this study. The cerclage was not replaced after dynamic loading to maintain imperfect cerclage tension and incomplete fracture reduction. According to the surgical guidelines final reaming should be 1.0 to 1.5 mm larger than the nail diameter to be used. Due to compressed and dense foam mimicking cancellous bone in the synthetic model, the intramedullary canal was reamed to 13 mm for the use of tibia nails with 11 mm diameter. The absence of muscles and soft tissue has been partly compensated for by applying a physiologic and clinically relevant load scenario of combined axial and torsional loads. Post-operative loading was simulated under moderate as well as full weight-bearing conditions to identify the stabilizing effect of additional cerclage wiring. The decision to investigate only steel cable cerclages is based on findings from the previous literature. Different cerclage materials have been tested in a biomechanical setup, revealing that steel cable cerclages show the largest reduction in interfragmentary motion [6,7].

## 5. Conclusions

In conclusion, from a biomechanical point of view, a well-reduced spiral fracture of the distal tibia is adequately stabilized by intramedullary nailing. Applying an additional cable cerclage increases shear stability, allowing the patient to bear full weight. In case of a larger fracture gap, additional cerclage wiring cannot adequately reduce interfragmentary movements. Therefore, post-operative rehabilitation should be in accordance with the type of fracture and the stability of the fixation. To further investigate the effect of additional cerclage wiring in distal tibia spiral fractures, further clinical trials are needed.

## Data Availability

Data relating to this study are available from the corresponding author upon reasonable request.
